# Incubation periods of enteric illnesses in foodborne outbreaks, United States, 1998–2013

**DOI:** 10.1017/S0950268819001651

**Published:** 2019-10-07

**Authors:** S. J. Chai, W. Gu, K. A. O'Connor, L. C. Richardson, R. V. Tauxe

**Affiliations:** 1 Division of Foodborne Waterborne and Environmental Diseases, Centers for Disease Control and Prevention, Atlanta, Georgia, USA; 2 Division of Global HIV and Tuberculosis, Centers for Disease Control and Prevention, Atlanta, Georgia, USA

**Keywords:** Epidemiology, food-borne infections, outbreaks

## Abstract

Early in a foodborne disease outbreak investigation, illness incubation periods can help focus case interviews, case definitions, clinical and environmental evaluations and predict an aetiology. Data describing incubation periods are limited. We examined foodborne disease outbreaks from laboratory-confirmed, single aetiology, enteric bacterial and viral pathogens reported to United States foodborne disease outbreak surveillance from 1998–2013. We grouped pathogens by clinical presentation and analysed the reported median incubation period among all illnesses from the implicated pathogen for each outbreak as the outbreak incubation period. Outbreaks from preformed bacterial toxins (*Staphylococcus aureus, Bacillus cereus* and *Clostridium perfringens*) had the shortest outbreak incubation periods (4–10 h medians), distinct from that of *Vibrio parahaemolyticus* (17 h median). Norovirus, salmonella and shigella had longer but similar outbreak incubation periods (32–45 h medians); campylobacter and Shiga toxin-producing *Escherichia coli* had the longest among bacteria (62–87 h medians); hepatitis A had the longest overall (672 h median). Our results can help guide diagnostic and investigative strategies early in an outbreak investigation to suggest or rule out specific etiologies or, when the pathogen is known, the likely timeframe for exposure. They also point to possible differences in pathogenesis among pathogens causing broadly similar syndromes.

## Introduction

Foodborne outbreaks are common in the United States. An average of ~1200 such outbreaks was reported annually during 1998–2008. Among these, about half lacked identification of a laboratory-confirmed etiologic agent [1]. Collecting appropriate clinical specimens and testing them with the appropriate tests remains a challenge, because of the wide array of pathogens, the difficulties of testing for all possibilities and the transient nature of the infection or intoxication. Identification of a laboratory-confirmed aetiology requires a series of conditions to be present, and gaps can preclude successful isolation and linking of an aetiology to the outbreak.

The incubation period is a fundamental characteristic specific to each pathogen that can provide a key piece of the epidemiological puzzle early in an outbreak investigation. If the pathogen is known, epidemiologists can use the incubation period to estimate a likely exposure period on which to focus interviews of cases and controls, make a case definition more precise and to suggest particular meals or other exposures on which to begin environmental evaluations. If a pathogen has not been determined, but the specific exposure is known, then investigators can use the measured incubation period, along with other clinical information, to hypothesize likely etiologies and focus diagnostic testing of clinical and food specimens on these etiologies [2].

Textbook descriptions of incubation periods for specific pathogens may provide little in the way of scientific documentation to justify them or are based on small series of outbreaks, and the incubation periods are often repeated from text to text. For example, a systematic review of incubation periods among enteric viruses found that only 50% of evaluated studies cited actual data, and of those which cited data, the majority of the data cited were traced back to a small number of original studies [3]. The incubation periods can also be determined from human volunteer challenge experiments in which the dose of the challenge strain can be controlled; however such trials are rare, expensive and usually limited to a single infecting strain [4].

To more systematically describe the distribution of incubation periods during foodborne outbreaks caused by common pathogens, we examined 16 years of outbreaks reported to national Foodborne Disease Outbreak Surveillance System (FDOSS).

## Methods

### Sources of data

The Centers for Disease Control and Prevention (CDC) collects data on foodborne disease outbreaks investigated and reported by U.S. states and territories through the FDOSS [1]. CDC defines a foodborne disease outbreak as the occurrence of two or more similar illnesses resulting from the ingestion of food in common. Reports include the etiologic agent, median incubation period of the illnesses, reported number of illnesses, frequency of symptoms, implicated food vehicle as well as other information. Salmonella isolates are serotyped in state public health laboratories using the Kauffman and White scheme [5] and serotype is reported. The median incubation period among the illnesses occurring in each outbreak is reported in minutes, hours or days. The reported data are an aggregated summary of the characteristics of ill persons involved in the outbreak, such as per cent affected, by sex; individual case data, including illness incubation period, are not reported.

### Statistical analysis

**Fig. 1.**
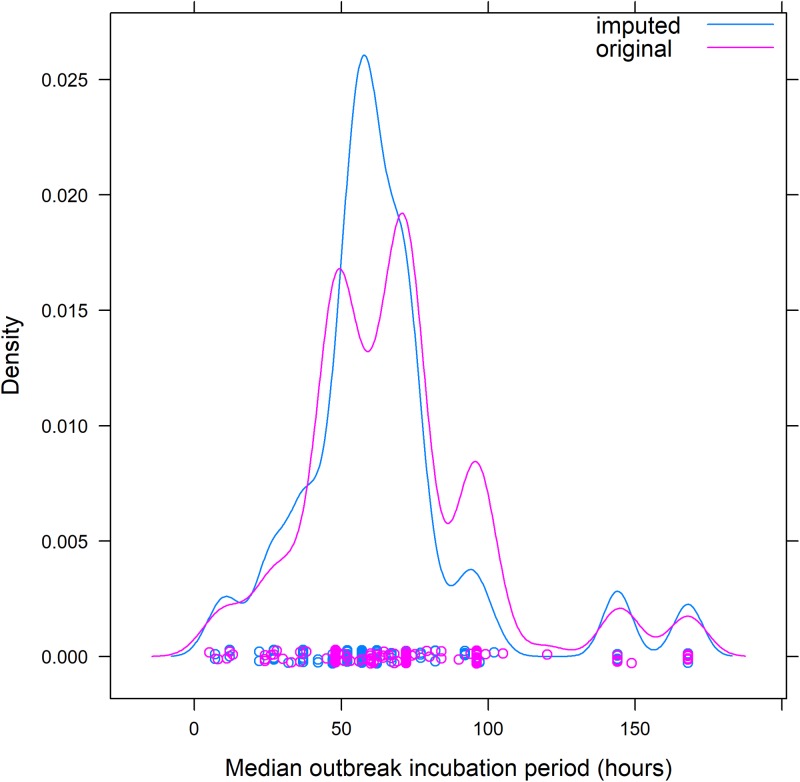
Example of redistribution of reported outbreak incubation periods* for foodborne outbreaks caused by *Campylobacter* spp. from dataset including days to dataset including only hours – Foodborne Disease Outbreak Surveillance System, 1998–2013. *Reported median of illness incubation periods within an outbreak.

We examined 16 years of foodborne disease outbreaks from laboratory-confirmed, single aetiology, enteric bacterial and viral pathogens reported to FDOSS from 1998–2013. To provide a stable estimate, we included pathogens that had caused more than 10 outbreaks. We excluded *Clostridium botulinum* as botulism has a substantially different clinical presentation than the other pathogens. As illness incubation periods of individuals are not reported to FDOSS, we analysed the reported median illness incubation period reported for each outbreak, hereafter termed ‘outbreak incubation period’, and excluded outbreaks with missing outbreak incubation period data. Most often, the outbreak incubation period was reported in hours. When the outbreak incubation period was recorded in minutes, we converted those to fractions of hours. Some outbreak incubation periods were reported in days; these data had less precision compared with outbreak incubation periods reported in hours, as outbreak incubation periods ⩾36 h and <60 h could be rounded to 2 days. To account for these less precise data, we imputed the incubation period for outbreaks reported in days into hours based on the distribution of outbreak incubation periods observed in other outbreaks of the same pathogen from 12 h before through 12 h after the reported number of days as the range in which rounding occurred. For example, 21 campylobacter outbreaks that reported an outbreak incubation period of 2 days (48 h) were imputed to bins between 36 and 60 h based on the observed frequencies of other campylobacter outbreaks that had outbreak incubation periods reported in hours ranging from 36 to 60 h. The imputed data smoothed arbitrary peaks at 24-h intervals introduced by inconsistent reporting of time units (Fig. 1).

We excluded outbreaks that had extreme values of incubation periods that were not biologically plausible based on the following criteria: (1) zero hours or zero days, (2) <5 h and >504 h (3 weeks) for nontoxin-producing bacterial pathogens and nonhepatitis viruses, (3) >24 h for *Staphylococcus aureus* and (4) >48 h for *Bacillus cereus* and *Clostridium perfringens*. We judged these reported outbreak incubation periods likely to be entry errors, and compared results before and after exclusion. We limited *Escherichia coli* outbreaks to those caused by Shiga toxin-producing *E. coli* (STEC) and *Vibrio* outbreaks to those caused by *Vibrio parahaemolyticus* because these were the most commonly reported pathotype or species of these two pathogen groups.

We examined outbreak incubation periods by the pathogen, and calculated a median for the group of outbreaks, and ranges that included 70% of the outbreaks (i.e. the 15^th^ through the 85^th^ percentile), hereafter called the ‘70% range’, and 95% of the outbreaks (i.e. the 2.5^th^ through the 97.5^th^ percentile), hereafter called the ‘95% range’. For each range we determined the width, defined as the time span between the longest and shortest outbreak incubation periods for each range.

We examined the relationship between outbreak size and outbreak incubation period variance to evaluate the need to weight outbreaks by size. Scatterplots of the reported outbreak incubation periods by the outbreak size did not reveal meaningful relationships between outbreak incubation period variance and outbreak size (Supplemental Figure), and the inverse-variance weighted medians and variances of outbreak incubation periods did not differ meaningfully from the unweighted estimates (data not shown). Therefore, we did not weight the outbreak incubation periods by the outbreak size for this analysis. We also considered whether age might affect incubation periods, but because age was reported inconsistently and only in aggregated age groups, and many outbreaks had a mixture of age groups, we judged the possibility of misattribution biases to be too high, so analyses by age were not conducted. Data on certain other factors with the potential to influence outbreak incubation periods, such as pathogen inoculum, were not reported to FDOSS.

As incubation periods can help distinguish illnesses with similar clinical presentations, we grouped pathogens based on typical clinical symptoms to conduct pairwise comparisons. We grouped all the bacterial infectious toxins and norovirus together because they typically present with a clinical syndrome of vomiting and diarrhoea in about 1 day, except *C. perfringens*, which typically presents with only diarrhoea; without diagnostic laboratory testing, these pathogens might be mistaken for one another. We also compared *Salmonella enterica*, *Shigella* spp. and *Campylobacter* spp. based on their typical clinical presentation of diarrhoea; we also included norovirus in this group, because of the overlap of its outbreak incubation period with these three bacteria. We also compared the four serotypes of salmonella most commonly reported among foodborne outbreaks [6].

All statistical comparisons were conducted on outbreak incubation periods that included imputed hours for outbreaks with outbreak incubation periods reported in days. We used the Mann–Whitney–Wilcoxon test (2-sided test, significance level of 0.05) to compare pairwise differences (including a Bonferroni correction) in the outbreak incubation period between pathogens and serotype using STATA statistical software version 13.0 (StataCorp, College Station, TX).

## Results

From 1998 through 2013, 6640 foodborne-disease outbreaks with a laboratory-confirmed bacterial or viral aetiology were reported to FDOSS. Of these, 6477 (98%) were associated with a single infectious aetiology, and of these, 4179 (65%) had an outbreak incubation period reported and were associated with an aetiology that caused more than 10 outbreaks. Most outbreaks with cases occurring in multiple states (92%) did not have an outbreak incubation period reported; among single-state outbreaks, a similar median and interquartile range of the number of ill persons were reported comparing outbreaks with and without an outbreak incubation period reported. We excluded 24 (1%) of the 4179 outbreaks based on biologically implausible outbreak incubation periods, leaving 4088 outbreaks to analyse further. No meaningful differences in outbreak incubation period results were noted before and after exclusion of these outbreaks. The number of ill persons per outbreak ranged from 2–1200 with a median of 15 and a mean of 29. Most (>99%) outbreaks affected people in only a single state and outbreaks generally had the same percentage of ill persons that were female as male (median of percentages reported by outbreak, 50% female; mean, 48% female).

Norovirus caused 53% of these outbreaks, followed by salmonella (23%). Eight other pathogens accounted for the remaining outbreaks: *S. aureus*, *B*. *cereus*, *C*. *perfringens*, *V. parahaemolyticus*, shigella, campylobacter, STEC and hepatitis A (Table 1).

**Table 1.** Reported outbreak incubation periods^a^ in foodborne outbreaks by aetiology, United States – Foodborne Disease Outbreak Surveillance System, 1998–2013

Aetiology	Outbreak incubation periods Median Hours	Median outbreak incubation period range 70% of outbreaks (15^th^–85^th^ percentile) Hours	Median outbreak incubation period range 95% of outbreaks (2.5^th^–97.5^th^ percentile) Hours	Outbreaks No. (%)
*Staphylococcus aureus*	4	2–5	2–8	153 (4)
*Bacillus cereus*	4	1.5–13.5	1–28	60 (1)
*Clostridium perfringens*	10	8–13	5–16	291 (7)
*Vibrio parahaemolyticus*	17	11–33	7–72	39 (1)
Norovirus	32	27–37	12–47	2172 (53)
*Salmonella enterica*	32	17–67	7–132	937 (23)
*Shigella* spp.	45	31–53	11–72	86 (2)
*Campylobacter* spp.	62	37–92	12–168	141 (3)
*Escherichia coli* (Shiga-toxin producing)	87	57–112	37–144	178 (4)
Hepatitis A	672	576–744	348–1008	31 (1)
Total				4088 (100)

a Reported median of illness incubation periods within an outbreak.

Similar median and 70% ranges of outbreak incubation periods were noted among groups of pathogens. Outbreaks caused by bacterial toxins preformed in food (*S*. *aureus* and *B*. *cereus*) or produced in the gut (*C*. *perfringens*) had the shortest median outbreak incubation periods, spanning 4–10 h, and similarly short 70% ranges, spanning 2–14 h as a group (Table 1). Seventy per cent of all outbreaks from *S*. *aureus* had outbreak incubation periods within a very narrow width of 3 h, whereas outbreaks from *B*. *cereus* had a 70% range with a width of 12 h. Similar to *S*. *aureus*, outbreaks due to *C*. *perfringens* had a narrow 70% range of 8–13 h with a width of 5 h, though the lower end of its 70% range of outbreak incubation periods started later, at 8 h.

*V. parahaemolyticus*, which does not cause illness from preformed toxins, had a median outbreak incubation period (17 h), a 70% range (11–33 h) and a 70% range width (22 h), that were for the most part, longer and distinct from those pathogens causing illness from preformed toxins.

A third group of pathogens, norovirus, salmonella and shigella, had similar incubation periods. Salmonella and norovirus had the same median outbreak incubation period (32 h). However, the 70% range for salmonella outbreaks (17–67 h) was wider and fully encompassed the narrow 70% range for norovirus outbreaks (27–37 h). The 70% range for shigella outbreaks (31–53 h) also fell within the 70% range for salmonella outbreaks but had a longer median outbreak incubation period (45 h). This 70% range width for shigella outbreaks was a little over twice that of norovirus (10 h), but less than half that of salmonella (50 h).

A fourth group of pathogens, campylobacter and STEC, had longer median outbreak incubation periods (62 and 87 h, respectively) that were greater than those of the other pathogens, except hepatitis A. The 70% range for campylobacter (37–92 h) overlapped with that for STEC (57–112 h), and about half of the campylobacter outbreaks (median outbreak incubation period of 62 h) had an outbreak incubation period greater than the lower end of the 70% range for STEC (57 h). However, about half of campylobacter outbreaks also had an outbreak incubation period less than the upper end of the 70% range for salmonella (67 h). The 70% range widths for campylobacter and STEC were the same (55 h) and were similar to those of salmonella (50 h).

Finally, hepatitis A had a median outbreak incubation period (672 h) nearly eight times longer than that of STEC, and its 70% range of outbreak incubation periods (576–744 h) was distinct from and much longer and wider (168 h) than all the other pathogens.

In pairwise comparisons between pathogens within clinical similarity groups, the outbreak incubation period distributions of the three short incubation toxin-producing bacterial pathogens and norovirus were significantly different from one another (all *P*-values <0.001, 6-comparison level of significance with Bonferroni correction is *P* < 0.008), except for the pair *B. cereus* and *S. aureus* (*P*-value = 0.21). Comparing norovirus, salmonella, shigella and campylobacter pairwise, we found a statistically significant difference between outbreak incubation period distributions for each pair (all *P*-values < 0.001), except salmonella and norovirus (*P*-value = 0.19)

**Fig. 2.**
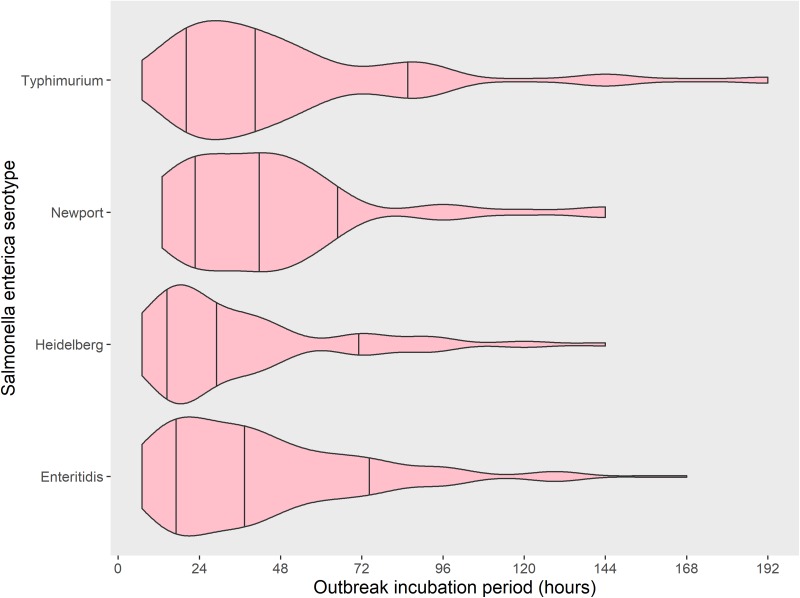
Distribution with median and 15^th^ to 85^th^ percentiles of reported outbreak incubation periods* of foodborne outbreaks caused by the four most commonly reported serotypes of *Salmonella enterica*, United States – Foodborne Disease Outbreak Surveillance System, 1998–2013. *Reported median of illness incubation periods within an outbreak.

The four most common *Salmonella enterica* serotypes caused 568 outbreaks, or 61% of the 937 *Salmonella enterica* outbreaks in this analysis: Enteritidis (318 outbreaks, 56%), Typhimurium (107, 19%), Heidelberg (82, 14%) and Newport (61, 11%). Distributions of the outbreak incubation periods showed a long right tail for all four serotypes. The incubation period of Heidelberg was shorter than the others (Table 2 and Fig. 2). Pairwise comparisons between Heidelberg and each of the other common serotypes either were significant (Typhimurium and Newport, both *P* < 0.008 using the Bonferroni correction) or trended towards significance (Enteritidis, *P* = 0.009). The incubation period distributions did not differ significantly between Enteritidis, Typhimurium and Newport (all pairwise comparison *P*-values ⩾ 0.15) (Table 3 and Fig. 2).

**Table 2.** Reported outbreak incubation periods^a^ of foodborne outbreaks caused by the four most commonly reported serotypes of *Salmonella enterica*, United States – Foodborne Disease Outbreak Surveillance System, 1998–2013

Serotype	Median outbreak incubation periods Median Hours	Median outbreak incubation period range 70% of outbreaks (15^th^–85^th^ percentile) Hours	Median outbreak incubation period range 95% of outbreaks (2.5^th^–97.5^th^ percentile) Hours	Outbreaks No. (%)
Enteritidis	37	17–72	7–127	318 (56)
Typhimurium	37	17–92	12–192	107 (19)
Heidelberg	22	17–72	7–120	82 (14)
Newport	41	19–65	13–144	61 (11)
Total				568 (100)

a Reported median of illness incubation periods within an outbreak.

**Table 3.** Mann–Whitney–Wilcoxon test of comparison *P*-values between reported outbreak incubation period^a^ distributions in foodborne outbreaks caused by the four most commonly reported serotypes of *Salmonella enterica*, United States – Foodborne Disease Outbreak Surveillance System, 1998–2013

Serotype	Typhimurium	Heidelberg	Newport
Enteritidis	0.152	0.009	0.480
Typhimurium		0.001	0.682
Heidelberg			0.007

a Reported median of illness incubation periods within an outbreak.

The 95% ranges of outbreak incubation periods can help exclude pathogens in an outbreak investigation. Using this criterion, a foodborne outbreak with an outbreak incubation period >8 h was unlikely to be caused by *S. aureus,* an outbreak with an outbreak incubation period >16 h was unlikely to be caused by *C*. *perfringens,* and an outbreak with an outbreak incubation period of >28 h was unlikely to be caused by *B. cereus*. Norovirus was suggested as a possible aetiology by an outbreak incubation period between 12–48 h, and was unlikely if the outbreak incubation was less than 12 h or more than 48 h. *V. parahaemolyticus* or shigella were unlikely etiologies if the outbreak incubation period was more than 72 h. Although the 95% ranges of salmonella and campylobacter overlapped substantially, salmonella had a median outbreak incubation period about half that of campylobacter. STEC was an unlikely aetiology if the outbreak incubation period was less than 37 h (about 1.5 days). Foodborne outbreaks with outbreak incubation periods >7 days were unlikely to be caused by the common bacterial and viral pathogens, except for hepatitis A (Table 1).

## Discussion

We quantified the substantial variation in median incubation periods during outbreaks caused by common foodborne pathogens. Approximate incubation periods are often available early in an outbreak investigation, and can help guide diagnostic and investigative strategies. The ranges we report may help suggest or rule out specific etiologies, or when the pathogen is known, the likely timeframe for exposure. They also point to possible differences in pathogenesis among pathogens causing broadly similar syndromes. The incubation period can differ even among salmonella serotypes, being shorter for Heidelberg than for three other common serotypes.

The three pathogens with the shortest outbreak incubation periods produce illness rapidly by elaborating toxins in the food before it is eaten (*S. aureus* and *B. cereus*) or soon after in the host (*C. perfringens*). While *S. aureus* and *C. perfringens* outbreaks can be readily distinguished on the basis of their median and 70% range of outbreak incubation periods, *B. cereus* causes two syndromes with outbreak incubation periods that overlap with the outbreak incubation periods of *S. aureus* and *C. perfringens*. Although we were not able to separate these two syndromes in our data, as only two outbreaks reported cases with only vomiting, the vomiting illness caused by *B. cereus* that produce the emesis toxin appears to be more common, resembles illness caused by *S. aureus* [7], and has a shorter outbreak incubation period. The diarrhoeal illness caused by *B. cereus* that produces the enterotoxin has a longer outbreak incubation period, is somewhat less common, and should be considered along with *C. perfringens* because of similar incubation periods and clinical syndromes [8].

Among the pathogens with intermediate length outbreak incubation periods, two groups can be identified, those with a shorter outbreak incubation period (*V. parahaemolyticus*, norovirus, salmonella and shigella) and those with a longer outbreak incubation period (campylobacter and STEC). This finding suggests that organisms may multiply at different rates in the host, and that the longer outbreak incubation period group may have more sequential pathogenic steps. *V. parahaemolyticus* multiplies rapidly in many media, and perhaps in the human gut as well, which might account for its relatively short median and 70% range of the outbreak incubation period. The median outbreak incubation periods for salmonella and norovirus are the same, but norovirus has a very narrow 70% range width of only 10 h, while that of salmonella is 50 h, suggesting there is a particular uniformity of pathogenesis for norovirus, while that of salmonella is likely to be affected by more factors. Notably, shigella has the longest outbreak incubation period of the group, and its pathogenesis via mucosal invasion differs from most of the pathogens in the longer incubation period group.

The wide range of outbreak incubation periods reported for salmonella likely reflects the effect of dose, food vehicle and host factors. This effect has been well demonstrated in outbreak settings where those consuming more of the implicated food became ill with salmonellosis more swiftly [9] and in *Salmonella* Typhi feeding trials, where the median incubation period was 5 days for those consuming 10^9^ organisms, and 9 days for those consuming 10^5^ organisms [10]. The implicated food vehicle also affects incubation periods, possibly because of the variation of pathogen concentration at a point of contamination or during food preparation [11]. It may also reflect variation in underlying medical conditions and host immunity [12], supported by the evidence that differences in the incubation period have been seen at the extremes of age [11]. More extreme incubation periods may occur. The exceptionally long incubation period documented in a raw milk-associated outbreak of *Salmonella* serotype Typhimurium infections (median, 8 days) remains well outside the 95% range for that serotype, and indicates that more needs to be learned about the determinants of incubation periods [13].

**Fig. 3.**
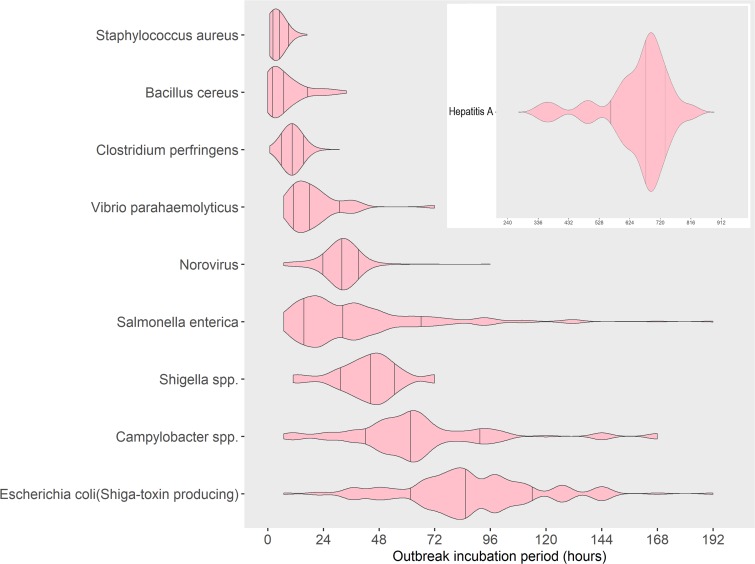
Distribution with median and 15^th^ to 85^th^ percentiles of reported outbreak incubation periods* in foodborne outbreaks by aetiology, United States – Foodborne Disease Outbreak Surveillance System, 1998–2013. *Reported median of illness incubation periods within an outbreak.

The shorter outbreak incubation period for outbreaks caused by *Salmonella* serotype Heidelberg could perhaps reflect variation in the speed of pathogenic progression or a difference in food vehicle and dose. It is possible that a difference in the bacterial metabolic state might account for some differences – serotypes with homeothermic food animal reservoirs like Heidelberg, Enteritidis and Typhimurium, may be preadapted to multiply more rapidly in the human gut, while those with poikilothermic reservoirs, like Newport, may need time to adjust to higher temperatures. Differences in temperature may result in a change in virulence – in *Salmonella* serotype Typhimurium, heat shock induces gene transcriptional changes that can increase virulence in the face of generic host defences, such as fever [14]. The decreasing length of median outbreak incubation periods by serotype (Fig. 3) follows the same order as the increasing per cent of foodborne outbreaks associated with animal food sources among the same serotypes (Newport 59%, Typhimurium 83%, Enteritidis 88% and Heidelberg 96%) [15]. Different serotypes that have different reservoirs may differ in the speed with which they infect and replicate in the human gut.

The incubation period for campylobacter is similar to but less variable than that of salmonella. In contrast to salmonella, no dose effect was demonstrated on the incubation period for *C. jejuni* in human volunteer experiments [16]. It has been suggested that younger age may be associated with a longer incubation period for outbreaks of campylobacter infections, though we were unable to assess the effect of age [17]. In a review of experimental studies and outbreaks involving campylobacter, median incubation periods (2 to 4 days) were found to be somewhat higher than in our study, perhaps owing to inclusion of cases without laboratory confirmation and non-foodborne outbreaks, of which the majority involved contact with a farm, commonly involved children, and had a 1.3 day longer average mean incubation period [17].

The median outbreak incubation periods and ranges can help during outbreak investigations, to suggest a potential pathogen, to define a period of interest within which to identify exposures, and when the pathogen is known, to guide the ramp down of enhanced surveillance for cases. Using a period of interest of 1 day before illness onset captures exposures for nearly all outbreaks caused by toxin-producing bacteria. A period of interest of 48 h before illness onset captures exposures in nearly all outbreaks caused by norovirus. Using 3 days before onset captures exposures in nearly all outbreaks caused by *V. parahaemolyticus* and shigella. For outbreaks caused by salmonella and campylobacter, the period of interest extends to 6 days before illness onset; the period includes 7 days for STEC, consistent with a review examining individual patients where all but one outbreak with an incubation period of >7 days were caused by animal contact, and not foodborne [18]. Capturing exposures for nearly all outbreaks of hepatitis A requires examining information spanning 2–6 weeks before the onset of illness, highlighting the difficulties of assessing exposures in hepatitis A.

Although these data are not based on individual cases, they suggest that different ranges may be optimal for conducting case-control studies of sporadic cases of different pathogens. The investigator can balance the more complete exposure assessment of using a longer period of interest against the lower quality of information collected when persons are asked to remember exposures from a longer time before their illness began. Examining a wider period of interest might not necessarily yield better results. In a Danish study of sporadic cases of *Salmonella* serotype Enteritidis infection, the results obtained varied by exposure window, and were most consistent when the exposure window was less than the maximum of 7 days [19].

While these data should be useful for planning investigations, they do not permit the absolute exclusion of possible exposures that occurred outside the 5–95% outbreak incubation period range. For each pathogen, outbreaks occur that have outbreak incubation periods outside that range, and while typographic or arithmetic errors in the reported information cannot be excluded, this variation can be quite real. Similarly, our data do not indicate the range of incubation periods of individual cases within the outbreak, and cannot be used to ‘prove’ that an individual case must be or cannot be part of a given outbreak. If the 95% outbreak incubation period range for *S. enterica* had been used in the Typhimurium outbreak among school children visiting a dairy farm, very few cases, if any, would have been considered part of the outbreak and the actual exposure would have been missed. As incubation periods of individual cases were not available, we used the reported median incubation period of each outbreak – i.e. the ‘outbreak incubation period.’ Since the variance of summarized data naturally depends on the number of observations in the dataset, the variance of the outbreak incubation periods for a pathogen and subsequently calculated median and ranges are potentially subject to bias arising from the distribution of outbreak sizes for that pathogen. Although our analyses examined and did not find biases requiring correction, if incubation periods of individual cases are available for future outbreaks, the extent of this potential bias can be better assessed.

Similar analyses examining outbreak incubation periods among waterborne outbreaks could provide important information to target exposure identification. When the mode of transmission for an outbreak is unknown at the time of investigation, considering differences in an outbreak incubation period for foodborne *vs.* waterborne transmission of the same pathogen or between different pathogens can be useful. Such analyses would also provide important information to target exposure identification for pathogens not occurring or occurring rarely in foodborne outbreaks, such as *Legionella* and *Cryptosporidium*. As greater numbers of outbreaks are investigated and reported over time and genotyping methods continue to develop, genotypic profiles of pathogens might be used to differentiate subtypes of pathogens that have different incubation periods, and perhaps different pathogenicity.

This analysis of outbreak incubation periods in foodborne outbreaks can provide outbreak investigators a useful tool to suggest possible aetiology and to help focus the time window to look for potential exposures. Differences among pathogens open questions regarding the effects of reservoir, dose, vehicle, pathogenicity and host. A confluence of epidemiological data, genotyping and other methods could be useful to explore answers to these questions further.

## Supplementary material

For supplementary material accompanying this paper visit https://doi.org/10.1017/S0950268819001651. click here to view supplementary material
