# Supplementary figures and images for: Incubation periods of enteric illnesses in foodborne outbreaks, United States, 1998–2013
Source: Epidemiol Infect. 2019 Oct 7;147:e285. doi: 10.1017/S0950268819001651 (PMC6805792; doi:10.1017/S0950268819001651)

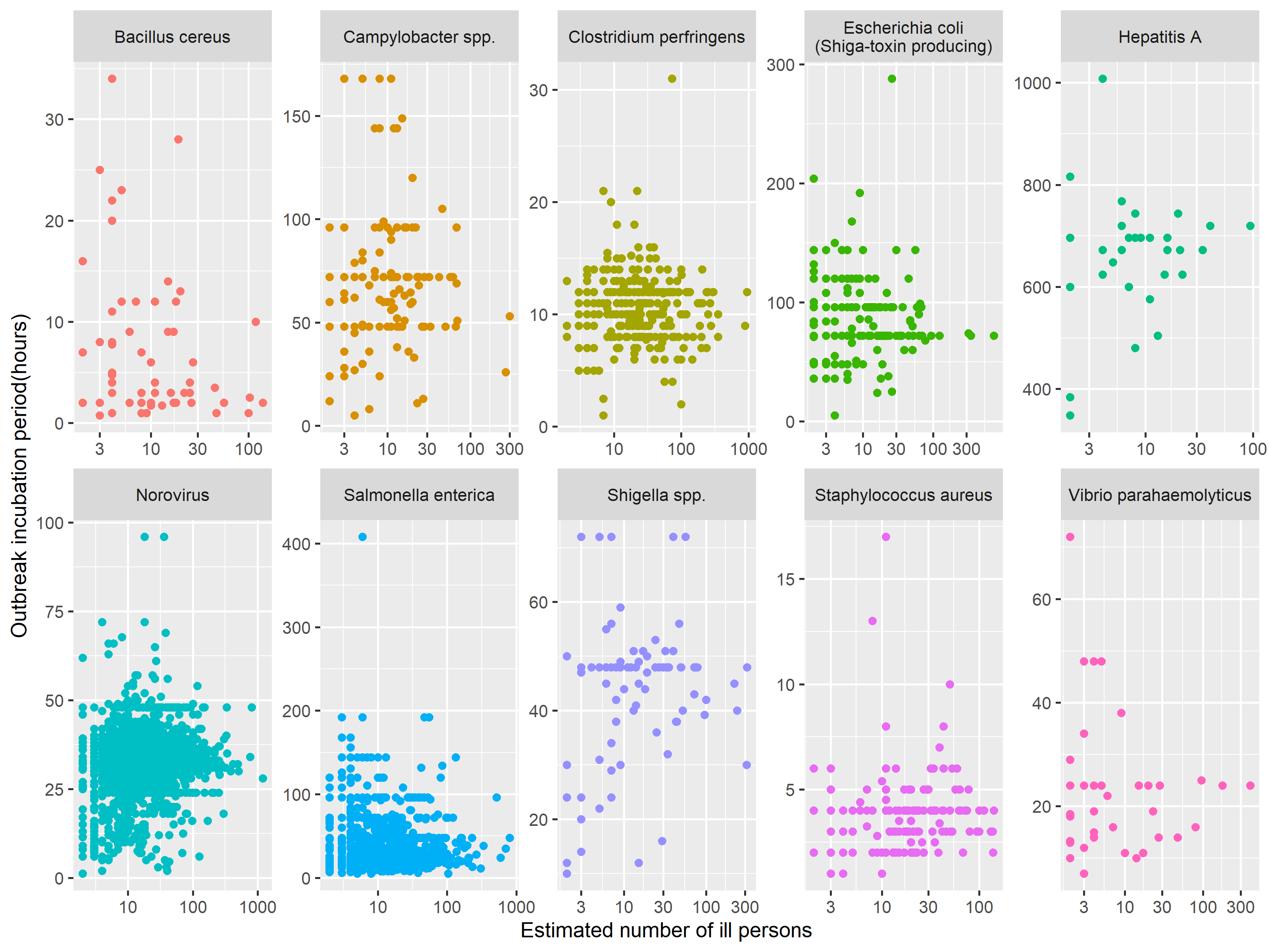

Supplement: Supplementary file 1 [file S0950268819001651sup001.png]
